# Modulation of Entrapment Efficiency and In Vitro Release Properties of BSA-Loaded Chitosan Microparticles Cross-Linked with Citric Acid as a Potential Protein–Drug Delivery System

**DOI:** 10.3390/ma13081989

**Published:** 2020-04-24

**Authors:** Natalia Sedyakina, Andrey Kuskov, Kelly Velonia, Nataliya Feldman, Sergey Lutsenko, Grigory Avramenko

**Affiliations:** 1 Department of Biotechnology, I.M. Sechenov First Moscow State Medical University (Sechenov University), Moscow 119991, Russia; nsedyakina@mail.ru (N.S.); n_feldman@mail.ru (N.F.); svlutsenko57@mail.ru (S.L.); 2 Department of Technology of Chemical Pharmaceutical and Cosmetic Substances, D. Mendeleev University of Chemical Technology of Russia, Moscow 125047, Russia; a_n_kuskov@mail.ru; 3 Department of Materials Science and Technology, University of Crete, 70013 Heraklion, Greece; velonia@materials.uoc.gr

**Keywords:** chitosan microparticles, citric acid, cross-linker, polyglycerol polyricinoleate, controlled release, protein delivery

## Abstract

Microparticles, aimed for oral protein and peptide drug delivery, were prepared via emulsion cross-linking using citric acid as cross-linker and polyglycerol polyricinoleate as surfactant. A comparative study of the interaction between chitosan and citric acid and its effect on the resulting microparticle properties was performed using different chitosan-to-cross-linker mass ratios and pH-values during fabrication of the microparticles. Non-cross-linked and cross-linked microparticles were studied in terms of size (4–12 μm), zeta potential (−15.7 to 12.8 mV), erosion (39.7–75.6%), a model protein encapsulation efficiency (bovine serum albumin) (6.8–27.6%), and loading capacity (10.4–40%). Fourier transform infrared spectroscopy and X-ray diffraction confirmed the ionic interaction between the protonated amine groups of chitosan and the carboxylate ions of the cross-linking agent. Scanning electron microscopy revealed that the non-cross-linked microparticles had an uneven shape with wrinkled surfaces, while the cross-linked formulations were spherical in shape with smooth surfaces. On the basis of these data, the role of the surfactant and microparticle structure on the release mechanism was proposed. Control of the microparticle shape and release mechanisms is expected to be crucial in developing carriers for the controlled delivery of proteins and peptides.

## 1. Introduction

Injection remains nowadays one of the most commonly used delivery routes for administering most protein and peptide drugs. However, it poses several disadvantages such as inconvenience to patients, need for several daily administrations owing to rapid elimination from circulation, risks of local allergy development followed by lipoatrophy, and rapid increase of protein concentration in the blood. At the same time, oral administration of protein and peptide drugs as an alternative to the injection route is hampered by their degradation in the harsh stomach environment; low permeability of the intestinal mucosa; and the presence of series of enzymes in the stomach, which results in protein denaturation and alters their bioactivity. Oral administration on the other hand offers improved patient compliance and decreased medical costs and, therefore, the development of efficient oral delivery systems for protein and peptide drugs is crucial.

Micro- and nano-scaled systems prepared from natural or synthetic polymers have recently found numerous applications in various biomedical and pharmaceutical fields, because their unlimited chemical diversity and their tunable particle size, size distribution, and morphology, as well as surface functionalities. Nano- and microparticles with entrapped therapeutic molecules, such as low molecular weight drugs, proteins, peptides, and DNA, are being actively studied owing to their potential to act as efficient drug delivery systems [1,2,3,4,5]. Colloidal micro- and nano-scaled delivery systems are of particular interest as their application has been proven to decrease undesirable drug toxic side effects and improve their bioavailability and therapeutic effect. The diverse delivery systems reported in the literature include liposomes, nanoparticles, microemulsions, and self-assembled polymeric nano and micro structures [6,7,8]. In this aspect, it has been previously shown that drug delivery systems based on amphiphilic poly-N-vinylpyrrolidone possess low toxicity, high biocompatibility, and stability, and can effectively entrap different low molecular weight and protein therapeutic agents [9,10,11,12,13].

Chitosan is a natural, linear glucosamine polymer mostly produced via an alkaline deacetylation of chitin [14]. Chitosan is non-toxic, mucoadhesive, and intrinsically biocompatible and biodegradable. Chitosan microspheres and microparticles are promising candidates for oral administration of biomacromolecules [15,16,17,18]. The free amino groups of chitosan promote cross-linking reactions, leading to the formation of chitosan three-dimensional network hydrogels [19]. The use of chitosan microspheres and microparticles for oral protein delivery not only allows the protection of the encapsulant from gastrointestinal conditions, but also ensures its prolonged release, which can be adjusted through swelling and degradation of the cross-linked polymer matrix in the small intestine. Nevertheless, several undesirable side-effects have been shown to arise from the use of chemical cross-linking agents, leading mostly to toxicity and deactivation of the encapsulated protein. To overcome such side-effects, a mild process of ionic cross-linking has been applied for the preparation of chitosan microspheres [20]. Ionotropic gelation methods take advantage of the electrostatic interaction between the positively charged protonated amino groups of chitosan and the negatively charged cross-linkers [21,22] such as, for instance, polyphosphates [23] and citrates [24].

The ability of the chitosan-based drug carriers to sorb and release guest active substances is significantly influenced by the conditions involved in the cross-linking process such as the chitosan-to-cross-linker ratio, pH, and temperature. Several literature reports have been devoted to chitosan films and gels cross-linked with citric acid that differ in terms of design and applications [25,26,27,28,29]. A limited number of literature reports focus on the effect of the conditions involved in the preparation process on the characteristics of the produced chitosan microspheres and microparticles cross-linked by the non-toxic and biocompatible citric acid. More specifically, Orienti et al. [30] obtained chitosan microspheres via thermal cross-linking in an inverse emulsion. In a similar system of citric acid cross-linked particles obtained by Varshosaz et al. [31] at a mild temperature, the effect of the cross-linking method on particle characteristics was presented.

Inspired by these early works, we previously proposed the use of a different surfactant to stabilize pre-emulsions [32], studied several dicarboxylic acid cross-linking agents [33], and investigated the effect of pH on the characteristics of citric acid cross-linked particles through ionotropic gelation [34].

Sorbitan monooleates or mixtures of sorbitan monooleates with polysorbates (Tween) are widely used as emulsifiers that stabilize pre-emulsions to prepare chitosan microspheres and microparticles [35,36,37]. Whereas in the case of thermal cross-linking, surfactant was not employed [30], Varshosaz et al. used sorbitan monooleate (Span 40) as emulsifier to stabilize pre-emulsion during non-thermal particle preparation [31]. Our previous studies revealed that polyglycerol polyricinoleates (PGPRs) can also be successfully employed as non-toxic, non-ionic surfactants to produce chitosan microparticles [32,33,34]. The effect of the chain length of dicarboxylic acids (malonic, succinic, glutaric, adipic, pimelic, suberic, azelaic) used to cross-link chitosan on the properties of the resulting chitosan microparticles was also investigated [33]. The properties of citric acid cross-linked chitosan particles produced using a pH 5.0 chitosan solution over a narrow range of polymer-to-cross-linker ratios was evaluated [34]. Via these sequentially performed, comparative studies [32,33,34], we were able to rationally improve the characteristics of chitosan particles as compared with those obtained in early works [31], and in particular, increase the encapsulation efficiency of biologically active substances and reduce the rate of its release.

Herein, we present a comparative study aimed to regulate the properties of chitosan microparticles and establish the optimal conditions for the preparation of these biocompatible drug carriers. We systematically varied all conditions that would influence the features of particles produced via citric acid chitosan cross-linking, that is, pH, polymer-to-crosslinker ratio, and temperature. The characteristics of cross-linked chitosan microparticles fabricated via a PGPR stabilized water/oil (W/O) emulsion were determined as means to control protein encapsulation efficiency and release ability aimed at oral protein and peptide drug delivery applications.

## 2. Materials and Methods

### 2.1. Materials

Eighty-two percent deacetylated chitosan with number-average molecular weight (MW) 200 kDa was purchased from Bioprogress (Shchyolkovo, Russia). Pionier^®^ liquid light paraffin oil was obtained from Hansen & Rosenthal Group (Hamburg, Germany). Polyglycerol-6-polyricinoleate (PG-6-PR, Hexaglyn PR-15, HLB 3.2) was purchased from Nikko Chemicals Co., Ltd. (Tokyo, Japan). Citric acid and bovine serum albumin (BSA) were purchased from Sigma-Aldrich (Sigma-Aldrich, St. Louis, MS, USA). All other chemicals and reagents were used as received.

### 2.2. Study of the Interaction between Chitosan and Citric Acid

Different concentrations of aqueous solutions of citric acid were added to chitosan solutions in a 2% w/v acetic acid solution (10 g/L) at mass ratios of chitosan-to-citric acid in the range of 0.2 to 5.0 using polymer stock solution with pH 4.0, 5.0, and 5.7. The samples were thermostated for three hours at 20 or 60 °C and stored at room temperature for several days. Separation of the system into two phases was visually observed and attributed to the formation of an insoluble chitosan-citrate complex.

### 2.3. Preparation of Chitosan Microparticles

To achieve microparticle formation, we optimized the emulsion cross-linking technique proposed by Varshosaz et al. [31]. More specifically, to prepare the aqueous phase, chitosan (200 mg) was initially dissolved in a 2% v/v aqueous solution of acetic acid (15 mL) at 50 °C. The pH of the polymer solution was adjusted to 5.0 or 5.7 with the addition of 1N NaOH solution. A 2.5% w/w polyglycerol-6-polyricinoleate solution in liquid paraffin (80 g) was used as the organic phase. The aqueous phase was added to the organic phase under magnetic stirring (200 rpm) at 60 °C and the resulting emulsion was then homogenized using an AM-11 Ace Homogenizer (Nihonseiki Kaisha Ltd., Tokyo, Japan) at 1500 rpm and room temperature for 5 min. The solution was then stirred using a magnetic stirrer (200 rpm) at 60 °C. After 1 h, 5 mL of a 0–10% w/w citric acid aqueous solution, acting as cross-linking agent, was dropwise added to the emulsion under stirring at at 60 °C for 5.5 h.

The resulting suspension of chitosan microparticles in paraffin oil was allowed to stand for five days to equilibrate. The top oil layer was decanted and the microparticles collected and washed 4–6 times with n-hexane by centrifugation at 6000 rpm for 10 min. After the final wash, the microparticles were allowed to dry in air. Finally, the particles were washed three times with 50 mL of dichloromethane and dried. Six different batches varying in the chitosan-to-cross-linker mass ratio were prepared at different pHs. More specifically, sample MP5704 was prepared using a chitosan-to-citric acid mass ratio of 0.4:1 (molar ratio 0.5:1) and pH 5.7; sample MP5706 with a mass ratio of 0.6:1 (molar ratio 0.7:1) and pH 5.7; and samples MP4008, MP5008, and MP5708 with a ratio of 0.8:1 (molar ratio 0.9:1) and pH 4.0, 5.0, and 5.7, respectively. Finally, a blank sample MP5700 was prepared without addition of the cross-linking agent (chitosan-to-citric acid ratio of 1:0) at pH 5.7.

### 2.4. Fourier Transform Infrared Spectroscopy (FT-IR)

FT-IR spectra were recorded by using a Spectrophotometer IR-380 (Nicolet, Thermo Electron Corporation, Waltham, MA, USA). Microparticle samples were measured in compressed KBr disks.

### 2.5. X-ray Diffraction Studies

Powder X-ray diffraction (XRD) using a STOE STADI-P diffractometer (STOE & Cie GmbH, Darmstadt, Germany) in transmission geometry employing Ge monochromated Cu K_a1_ radiation, whose wavelength was 1.54 Å, was used to characterize both the non-cross-linked and cross-linked chitosan microparticles. The patterns were indexed and unit cell parameters were determined from least squares refinement.

### 2.6. Surface Morphology and Particle Size

Scanning electron microscopy (raster electronic microscope MIRA 3, Tescan, Czech Republic) at an accelerating voltage of 1 kV was used to determine the shape, surface morphology, and particle size of the chitosan microparticles. Prior to analysis, the samples were dried under vacuum. The diameters of not less than 300 microparticles were randomly measured and the mass-average diameters of the particles (D_m_,µm) were calculated using Equation (1):

(1) $$D_{m}(µm)=\frac{\sum n_{i}D_{i}^{4}}{\sum n_{i}D_{i}^{3}}$$

where n_i_ is the number of particles with D_i_ as diameter (µm).

### 2.7. Zeta Potential

Zeta potential of the chitosan microparticles was determined using a Zetasizer Nano ZS (Malvern Instruments Ltd., Malvern, UK). The samples were prepared according to the method reported by Zhang et al. [38].

### 2.8. Erosion Studies

Accurately weighed unloaded microparticles (10 mg) were incubated in phosphate buffer saline (pH 7.4) at room temperature for 48 h prior to erosion studies. Upon collection, the microparticles were washed twice by centrifugation with distilled water at 6000 rpm for 10 min, dried in the oven at 40 °C for 24 h, and reweighed to determine erosion.

The following equation was used to determine erosion:

(2) $$\mathrm{Erosion}\;(\%)=\frac{w_{0}-w_{e}}{w_{0}}\times 100\%$$

where w_0_ is the weight of unloaded dry microparticles (mg) and w_e_ is the weight of the eroded dry microparticles (mg).

### 2.9. Microparticle Loading—Determination of Encapsulation Efficiency (EE) and Loading Capacity (LC)

The chitosan microparticles were loaded with bovine serum albumin (BSA) via the adsorption method. More precisely, 12 mL of a 4.0 g/L BSA solution in PBS, pH 7.4, was mixed with 30 mg of dried microparticles. The suspension was kept for 48 h to allow loading of the albumin by adsorption. The resulting loaded microparticles were subsequently recovered by centrifugation and dried in a desiccator at room temperature. The residual BSA present in the supernatant was determined by the Bradford protein assay [39]. From the results obtained via the Bradford assay, the encapsulation efficiency (%) and the loading capacity (%) were calculated using the following equations:

(3) $$\mathrm{EE}\left(\%\right)=\frac{\mathrm{Initial}\;\mathrm{weight}\;\mathrm{of}\;\mathrm{BSA}\;-\;\mathrm{Weight}\;\mathrm{of}\;\mathrm{BSA}\;\mathrm{in}\;\mathrm{supernatant}}{\mathrm{Initial}\;\mathrm{weight}\;\mathrm{of}\;\mathrm{BSA}}\times 100\%$$

(4) $$\mathrm{LC}\left(\%\right)=\frac{\mathrm{Initia}\;\mathrm{Weight}\;\mathrm{of}\;\mathrm{BSA}\;-\;\mathrm{Weight}\;\mathrm{of}\;\mathrm{BSA}\;\mathrm{in}\;\mathrm{supernatant}}{\mathrm{Weight}\;\mathrm{of}\;\mathrm{the}\;\mathrm{microparticles}}\times 100\%$$

### 2.10. In Vitro Release Study

The microparticles (10 mg) were placed in 2 mL PBS solution, pH 7.4, and incubated at 37 °C in a thermostated shaker (100 rpm). At predetermined time intervals, the supernatant was collected after centrifugation at 6000 rpm for 5 min and replaced with the same volume of fresh PBS [15]. The amount of BSA released from the microparticles was determined by the Bradford protein assay and expressed as a percentage of total BSA entrapped in the samples.

### 2.11. Statistical Analysis

Each assay was repeated three times. Statistical significance was expressed as mean ± standard deviation (mean ± S.D.). The results were analyzed with one- and two-way analysis of variance (ANOVA) tests. The differences were considered to be significant at a level of *p* < 0.05.

## 3. Results and Discussion

### 3.1. Study of the Interaction between Citrate and Chitosan

**Figure 1:**
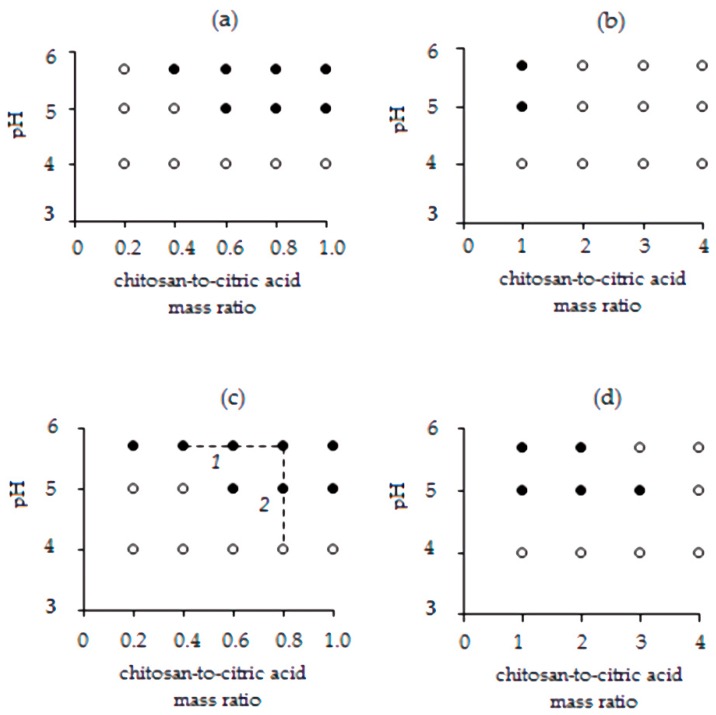
The effect of the chitosan-to-citric acid mass ratio and of pH of the polymer stock solution on the formation of the chitosan–citric acid complex at 20 °C (**a**,**b**) and 60 °C (**c**,**d**). Filled symbols (●) denote phase-separated systems, and open symbols (○) denote homogeneous systems.

The interaction between citric acid and chitosan was investigated at a high polymer solution concentration (10 g/L) at 20 °C. Conveniently, the formation of a two phase-system, attributed to the formation of an insoluble complex between chitosan and citrate, could be visually assessed. As shown in Figure 1a,b, no chitosan–citrate complex could be visually observed at a low pH (4.0) of the chitosan stock solution.

In this pH, a low degree of ionization of the carboxyl groups of the citric acid is expected, whereas more than 90% of the amine groups of chitosan are expected to be protonated [24]. As a result, the electrostatic interaction between the polymer and the cross-linker is expected to be weak, not leading to the formation of an insoluble complex. At pH 5.0 and 5.7, а white flocculent precipitate was observed, indicating the formation of a chitosan–citrate complex owing to the increased charge density of citrate [24]. Figure 1a,b present the full range of component ratios found to afford the formation of an insoluble complex. It was shown that the increase of chitosan solution pH from 5.0 to 5.7 resulted in complex formation even at a lower chitosan to citric acid mass ratio.

To obtain data relevant to the evaluation of chitosan drug carriers, this study was carried out under the conditions used for the preparation of chitosan microparticles, that is, using a chitosan solution concentration of 10 g/L at 60 °C (Figure 1c,d). The increase in temperature used for the preparation of the complex from 20 to 60 °C widened the range of component ratios at which an insoluble complex could be formed. Our findings clearly demonstrated that all three factors, that is, increase of pH, increase of temperature, and concentration ratios, can be tuned in order to promote the formation of the chitosan–citric acid complex. The pronounced effect of temperature can be attributed to the rupture of hydrogen bonds within the macromolecule of chitosan at 60 °C. This results in a larger number of amino groups available for interaction with the negatively charged carboxyl groups of citric acid. This assumption is supported by the findings of Krayukhina et al. reporting that the interaction of chitosan with a hydrophilic copolymer of maleic acid and N-vinylpyrrolidone at an elevated temperature (70 °C) resulted in the formation of a polyelectrolyte complex saturated with chitosan macromolecules owing to the rupture of weak intermolecular bonding caused by heat treatment [40].

On the basis of the results of this study, different chitosan-to-cross-linker mass ratios and pH-values (lines 1 and 2, Figure 1c) were selected for the preparation of the chitosan microparticles and subsequent investigation of their properties. Unstabilized (not cross-linked) microparticle samples of chitosan were also fabricated in the absence of a citric acid under the same conditions.

### 3.2. Characterization of Microparticles

Ionically cross-linked chitosan microparticles were successfully prepared via the emulsification-cross-linking technique, using polyglycerol polyricinoleate (PGPR) as surfactant and citric acid as cross-linking agent [30]. Briefly, stock chitosan solutions (pH 4.0, 5.0 or 5.7) were added to a liquid paraffin solution at 60 °C under vigorous stirring. The suspensions were homogenized and equilibrated under stirring at 60 °C, prior to the addition of an aqueous citric acid solution. Upon equilibration, the microparticles were separated and washed several times prior to drying. On the basis of the visual assessment of complex formation (3.1), six different batches varying on the pH and the chitosan-to-cross-linker mass ratio were prepared. Namely, sample MP5704 was prepared using a pH 5.7 chitosan stock solution and a chitosan-to-cross-linker mass ratio of 0.4:1, and sample MP5706 at pH 5.7 and ratio of 0.6:1, sample MP5708 at pH 4 and ratio of 0.8:1, sample MP5008 at pH 5 and ratio of 0.8:1, and sample MP4008 at pH 4.0 and ratio of 0.8:1, respectively. Finally, at pH 5.7 and a chitosan-to-cross-linker mass ratio of 1:0, the non-cross linked sample MP5700 was prepared. The microparticles were analyzed for different physico-chemical properties to comparatively evaluate the effect of the preparation conditions on their ability to form stable drug delivery systems.

#### 3.2.1. Fourier Transform Infrared Spectroscopy (FT-IR)

**Figure 2:**
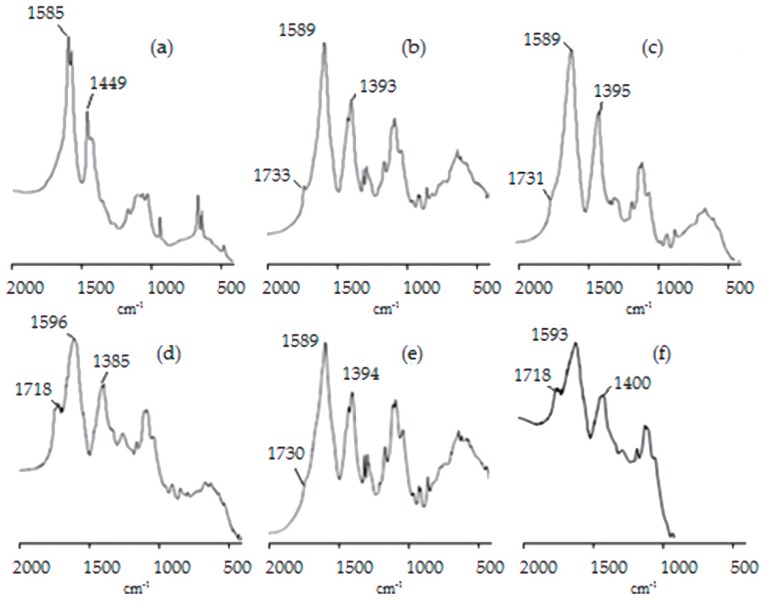
Fourier transform infrared spectroscopy (FT-IR) spectra of the non-cross-linked MP5700 (**a**) and cross-linked MP5708 (**b**), MP5706 (**c**), MP5704 (**d**), MP5008 (**e**), and MP4008 (**f**) chitosan microparticles.

FT-IR spectra of the chitosan microparticles are presented in Figure 2. The FT-IR spectrum of the non-cross linked sample (MP5700) revealed a distinct peak at 1585 cm^−1^, corresponding the N–H bending vibration of the free amino group of chitosan [41]. The bending vibrations of C–H and O–H in the ring of chitosan were detected at 1448 cm^−1^. Three stretching vibrations at 1153, 1046, and 1013 cm^−1^ could be attributed to the C–O–C, С–О, and C–C bonds, respectively [42].

The FT-IR spectra of the cross-linked samples (Figure 2b–f) presented two new characteristic peaks at ~1730 cm^−1^ attributed to the C=O stretching vibrations of the carboxylic moiety and at ~1390 cm^−1^ attributed to symmetric stretching vibrations of the carboxylate ion. Both new bands are indicative of the presence of citrate within the chitosan matrix, providing support of the ionic interaction between the positively charged protonated amine groups of chitosan and the negatively charged carboxylate ions of the citric acid [26].

#### 3.2.2. X-ray Diffraction (XRD)

The X-ray powder diffraction patterns of the samples are shown in Figure S1a–c. The XRD of non-cross-linked sample MP5700 showed various narrow and sharp peaks between about 8° and 70°, indicating the crystalline nature of the microparticles (Figure S1a). Among them, two peaks at around 12° and 19° are characteristic of semi-crystalline chitosan [27]. For all cross-linked microparticles, with the exception of the sample MP5704, most of the sharp peaks disappeared, the characteristic peaks of the chitosan became wider and weaker, and a new peak was observed at 37°. These changes demonstrated that the crystallinity of the batches decreased, a fact that can be attributed to the strong interaction of chitosan with citric acid, which impairs the molecular order within the particles [28,29]. The X-ray diffraction pattern of MP5704 was closest to that of the non-cross-linked sample, indicating a low degree of polymer cross-linking. The increase in the chitosan-to-citric acid ratio led to a reduction of the intensity of the peak at 19°, which indicates an increase in the cross-linking density of the microparticles (Figure S1a–c). A broadening of the peak at 19° and a decrease in its intensity in the order MP4008, MP5008, and MP5708 (Figure S1c) was also observed. Thus, the sample MP5708 showed the highest cross-linking density of chitosan compared with other samples under study.

#### 3.2.3. Scanning Electron Microscopy (SEM)

**Figure 3:**
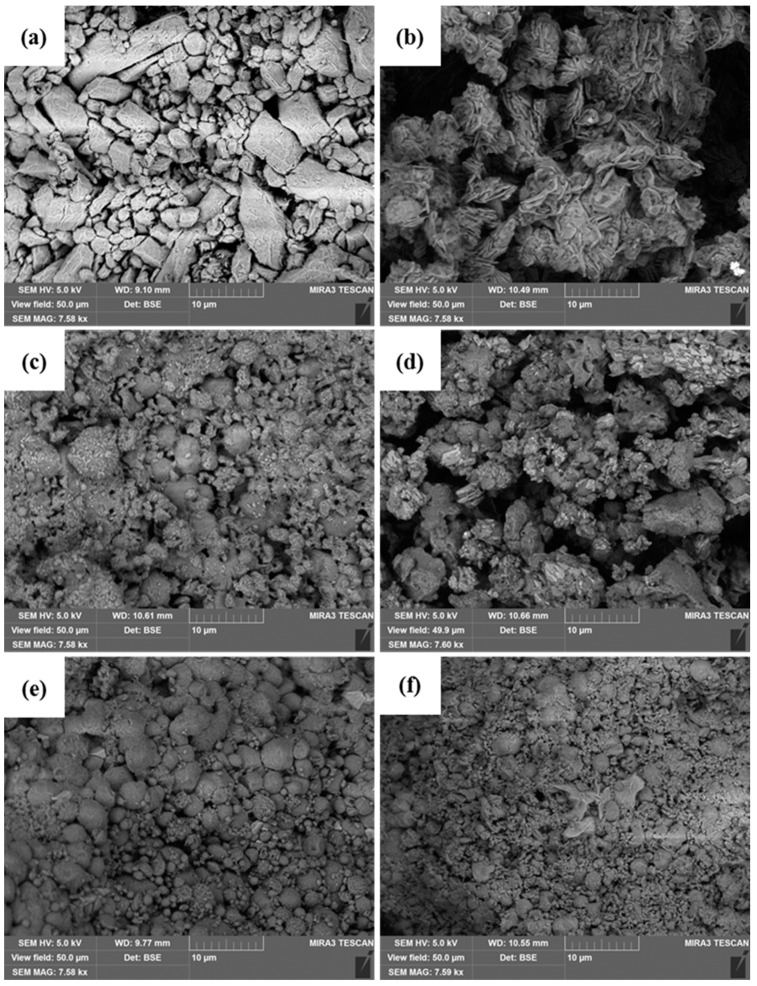
Scanning electron microscopy (SEM) microphotographs of the non-cross-linked MP5700 (**a**) and cross-linked MP5704 (**b**), MP5706 (**c**), MP5708 (**d**), MP5008 (**e**), and MP4008 (**f**) chitosan microparticles.

Scanning electron microscopy was used to visualize the shape and surface morphology of the chitosan microparticles (Figure 3). Aggregation of the obtained microparticles was observed. The non-cross-linked sample MP5700 presented an uneven shape with a wrinkled surface, while all formulations prepared in the presence of CA were nearly spherical in shape with a relatively smooth surface. The different morphologies support the chitosan–citric acid ionic cross-linking interaction. Furthermore, the needle-like crystals in sample MP5704 are indicative of the precipitation of the excess of citric acid during the preparation of the particles and could indicate adsorption on the surface of the particles owing to van der Waals interactions.

#### 3.2.4. Size and Zeta Potential of Chitosan Microparticles

**Figure 4:**
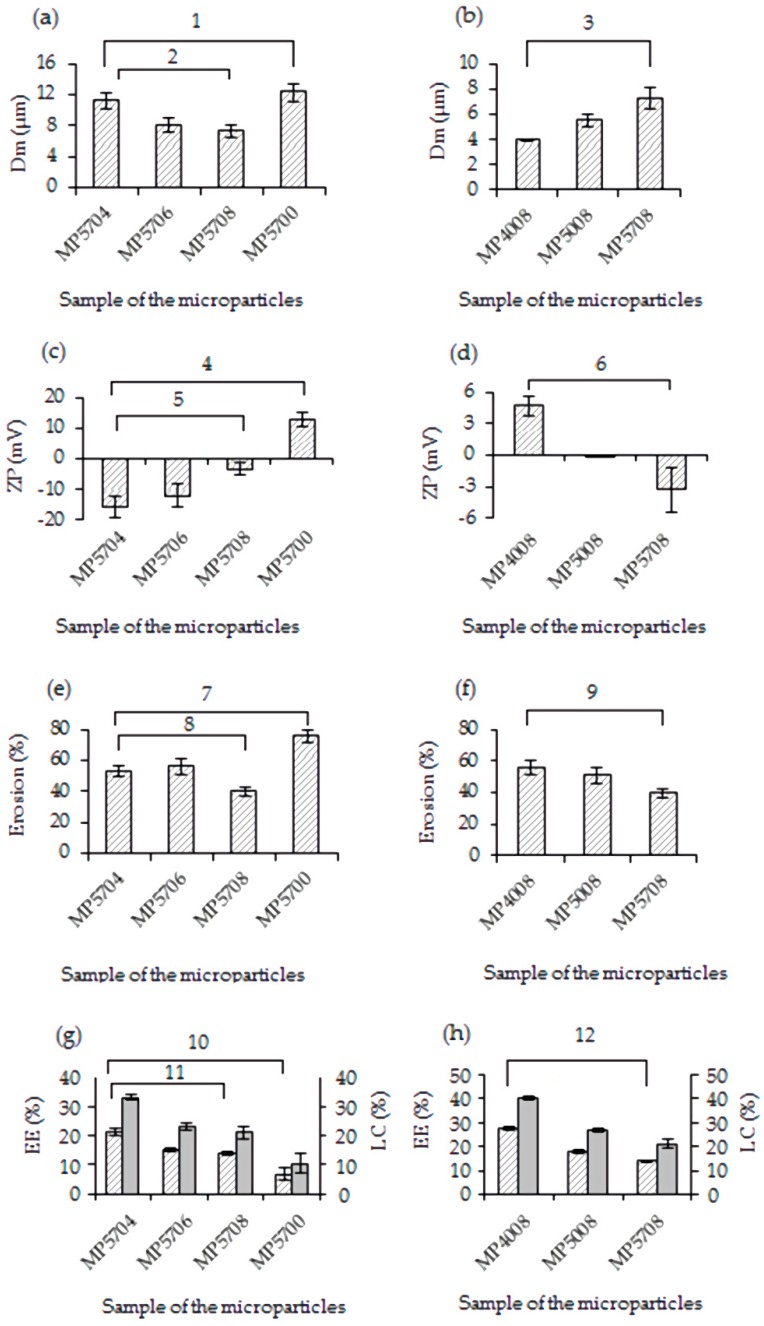
D_m_ (**a**,**b**), zeta potential (ZP) (**c**,**d**), erosion (**e**,**f**), encapsulation efficiency (EE), and loading capacity (LC) (**g**,**h**) for the non-cross-linked and cross-linked chitosan microparticles prepared at different polymer-to-crosslinker ratios (**a**,**c**,**e**,**g**) and pH values (**b**,**d**,**f**,**h**). The *p*-values (1—8.48 × 10^−4^; 2—5.26 × 10^−3^; 3—1.14 × 10^−3^; 4—2.1 × 10^−7^; 5—2.72 × 10^−3^; 6—2.13 × 10^−3^; 7—1.59 × 10^−7^; 8—1.33 × 10^−3^; 9—4,15 × 10^−3^; 10—6.92 × 10^−6^; 11—9.83 × 10^−5^; 12—4.78 × 10^−7^) were obtained from the one-way analysis of variance (ANOVA).

The mass-average particle size of the microparticles was determined by SEM. All microparticles were visualized to possess mean diameters significantly lower than 12 μm (Figure 4a–d). Compared with non-cross-linked sample (D_m_ of 12.3 μm), the decrease of the average size of the microparticles prepared with citric acid is presumably a result of a tightening effect caused by the cross-linker. At the same pH (5.7), the D_m_ of the microparticles decreased from 11.2 to 7.3 with the decrease of the concentration of citric acid in the aqueous phase in the order MP5704 > MP5706 > MP5708 (one-way analysis of variance (ANOVA) test, *p* < 0.05, Figure 4a). This is indicative of the increase in the cross-linking density of the chitosan matrix. At the same time, the mass-average particle size increased with pH at the same chitosan-to-citric acid ratio when comparing samples MP4008, MP5008, and MP5708 (Figure 4b).

The zeta potential (ZP) values of the microparticle preparations were in the range of −15.7 to 12.8 mV (Figure 4c,d), demonstrating that the particles are not able to form stable suspensions and tend to aggregate. The data obtained from the zeta potential measurements are in full agreement with the results of the microscopic study (Figure 3).

More specifically, the non-cross-linked sample surface was positively charged owing to the protonated amine groups of chitosan. The addition of citric acid in the formulations resulted in reduction of the zeta potential as compared with that of MP5700, a fact that could be attributed to cross-linking. The decrease in the ZP values with the decrease of the chitosan-to-citric acid mass ratio in the sequence of MP5708, MP5706, and MP5704 (one-way ANOVA test, *p* < 0.05) can be attributed to an increase in the excess of citrate adsorbed on the surface of the particles. A decrease in ZP values in the order MP4008, MP5008, and MP5708 (Figure 4d) was also observed (one-way ANOVA test, *p* < 0.05). This can be attributed to the deprotonation of positively charged amine groups and ionization of the carboxylic groups of the cross-linker with the increase of pH, leading to an overall change of charge at the particle surface.

#### 3.2.5. Erosion of the Microparticles

The erosion behavior of the chitosan microparticles with respect to weight loss in phosphate buffer saline was determined (Figure 4e,f). The microparticles prepared with citric acid exhibited the lower degree of degradation (40–56%) in comparison with that of sample MP5700 (about 76%). This can most probably be attributed to the formation of the ionically cross-linked chitosan matrix. The decrease of cross-linking agent concentration in the aqueous phase led to a decrease in the percentage of erosion in the order MP5704, MP5706, and MP5708 (one-way ANOVA test, *p* < 0.05) (Figure 4e), owing to the growth of density of the three-dimensional network of the particles. In the presence of an excess of citric acid, the number of amino groups that can interact with citrate decreases owing to their protonation. Therefore, the degree of cross-linking at a ratio of chitosan-to-citric acid of 0.4:1 is lower than at 0.8:1.

The degree of erosion when varying the pH in the series MP4008, MP5008, and MP5708 decreases from 56% to 40% (one-way ANOVA test, *p* < 0.05) (Figure 4f). Hence, it is clear that the cross-linking density of the microparticles is influenced by pH value owing to changes in the degree of ionization of the cross-linker.

### 3.3. Encapsulation Efficiency and Loading Capacity

As shown in Figure 4g, the BSA encapsulation efficiency (EE) and loading capacity (LC) of the microparticles decrease with the increase of the chitosan-to-citric acid ratio (one-way ANOVA test, *p* < 0.05) from 21.4% to 13.8% and from 33% to 21%, respectively. According to Figure 4h, a pH increase results in a decreased percentage of BSA encapsulation from 27.6% to 13.8% and of BSA loading capacity from 40.3% to 21% (one-way ANOVA test, *p* < 0.05). These results are in good agreement with data on the dependence of the cross-linking density of the microparticles on the same factors. High density of cross-linking of the chitosan causes steric hindrance, preventing protein from penetrating into the polymer matrix, and thus reducing BSA encapsulation efficiency. A lower cross-linking density allows more BSA molecules to be encapsulated per particle.

The low entrapment efficiency and loading capacity of albumin in sample MP5700 (6.8% and 10.4%, respectively) could be associated with a high degree of degradation of the non-cross-linked microparticles.

### 3.4. In Vitro BSA Release

**Figure 5:**
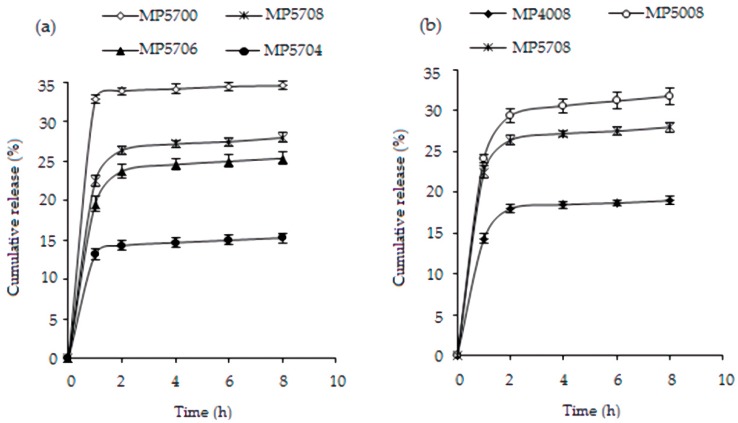
The effect of the chitosan-to-citric acid mass ratio (**a**) and pH value of the polymer stock solution (**b**) on the in vitro cumulative release of bovine serum albumin (BSA) from the non-cross-linked and cross-linked chitosan microparticles.

The cumulative BSA release profiles are depicted in Figure 5. In all cases, the initial burst release of the protein (within the first one to two hours) governed by the desorption of the protein molecules associated with the surface of the microparticles was followed by a stage of slower release that can be attributed to swelling of the chitosan matrix and diffusion of the protein from the micropores of the particles [43]. BSA release is shown to be dependent on the degree of cross-linking of the chitosan matrix. Longer initial release periods are achieved for the cross-linked microparticles as compared with the non-cross-linked sample MP5700. The latter demonstrated the highest percentage of the initial release of the protein of 33.6% (two-way ANOVA test, *p* < 0.05). The initial protein release increased in the order MP5704, MP5706, and MP5708 from 13.2% to 26.4%, that is, with the increase of the cross-linking density of the microparticles (two-way ANOVA test, *p* < 0.05) (Figure 5a). This can be probably attributed to the fact that a high degree of cross-linking would obstruct the penetration of BSA adsorbed on the particle surface during protein sorption into the chitosan matrix, leading to a desorption of a greater amount of the surface bound protein drug during the initial release stage.

The cumulative release of BSA from sample MP4008 exhibited a burst release of 18%, which is significantly lower than that from MP5008 and MP5708 (29.4% and 26.4%, respectively; two-way ANOVA test, *p* < 0.05, Figure 5b). The lower cross-linking density of sample MP4008 can be related to increased diffusion of the adsorbed albumin from the particle surface toward its core as compared with the more tightly cross-linked samples. As a result, a lower amount of BSA would be exposed in the surface layer and released into the buffer during the initial burst. The ionic interactions formed between the positively charged protonated amino groups of the chitosan matrix of MP4008 and the negatively charged groups of the molecules of the entrapped protein resulted in a better retention of the albumin by the particles and led to a reduced percentage of initial release of the loaded protein.

### 3.5. Role of the Surfactant and Proposed Structure of the Microparticles

**Figure 6:**
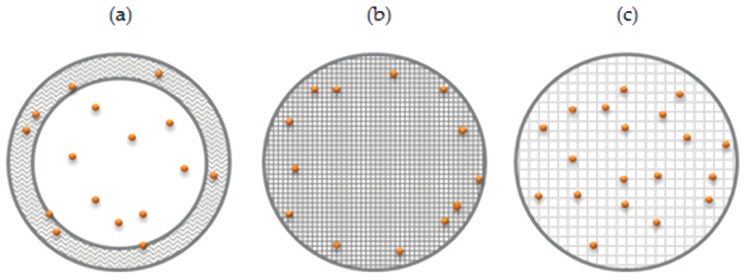
Schematic representation of proposed microparticle structures and BSA (orange dots) loading patterns: microparticles with core–shell structure (**a**), microparticles possessing a high degree of cross-linking of the polymer matrix (**b**), and microparticles possessing a lower degree of cross-linking of the polymer matrix (**c**).

Figure 6 schematically describes the proposed structures of the BSA-loaded microparticles.

The textural properties of the particles were indirectly evaluated by the determination of the degree of sorption of the biologically active substance (BSA) in the particles and assessment of the BSA release profiles. The results of this study were in good agreement with data obtained on the dependence of cross-linking density of the microparticles on the chitosan-to-citric acid ratio and the pH of the chitosan stock solution. On the basis of the obtained results, we can safely assume the following mechanism of BSA encapsulation by the cross-linked chitosan microparticles: at the first stage of sorption, protein molecules were adsorbed on the surface of the particles; at the second stage of this process, a fraction of the protein molecules located near the particle surface diffused into the polymer matrix. High density of chitosan cross-linking causes steric hindrance, preventing the protein from penetrating into the polymer matrix, and thus reducing BSA encapsulation efficiency. A lower cross-linking density allows more BSA molecules to be encapsulated per particle.

The initial burst release of the protein from the microparticle samples is governed by the desorption of the protein molecules associated with the surface of the microparticles at the first stage of BSA release. The slower release rate observed for the second stage can be attributed to swelling of the chitosan matrix and diffusion of the protein from the micropores of the particles, determined by the degree of cross-linking of the polymer.

On the basis of the obtained results, we can safely assume the existence of hydrophobic interactions and the formation of hydrogen bonds between molecules of the surfactant and chitosan. We have previously shown that mixtures of PG-6-PR-chitosan exhibit synergism in the efficiency of the surface tension reduction [33]. As a result, hydrophobic layers should be formed on the surface of the dispersed phase drops of the pre-emulsion in the absence of a cross-linking agent. Hence, it can be assumed that the sample MP5700 formed a core–shell structure composed of a polyelectrolyte-rich shell and a polyelectrolyte-poor core (Figure 6a). Previously, Grant et al. [44] showed a similar structure prepared using mixtures of chitosan and sorbitan monolaurate without a cross-linker. The sample MP5700 was found to possess a low percentage of entrapped protein and result in a rapid initial BSA release owing to significant destruction of its non-stabilized shell.

The cross-linked samples are in fact polyelectrolyte gel microparticles (Figure 6b,c). The high degree of cross-linking of samples MP5708 and MP5008 created steric hindrance for the diffusion of BSA molecules into the chitosan matrix (Figure 6b). As a result, most of the protein molecules were most probably located near the particle surface and the microparticles exhibited the highest initial release of the BSA. The sample MP5704 possessed a lower degree of cross-linking and the protein molecules diffused more easily into the particles and were trapped by the polymer matrix (Figure 6c). For this reason, the microparticles demonstrated a lower percentage of release in the initial phase. Most probably, the structure of microparticles formed by sample MP5706 occupied an intermediate structure between that of MP5704 and of MP5708.

The sample MP4008 should possess a structure similar to that shown in Figure 6c, with the albumin molecules positioned in the interior of the chitosan matrix of MP4008 owing to the low degree of cross-linking of the microparticles. The high density of the positive charge of protonated chitosan would support ionic interaction of BSA with the polymer and its retention within the particles. As a result, a low percentage of initial release of protein into the buffer medium was observed.

## 4. Conclusions

Ionically cross-linked chitosan microparticles were successfully prepared via the emulsification-cross-linking technique using polyglycerol polyricinoleate (PGPR) as surfactant and citric acid as cross-linking agent. Imaging of the chitosan microparticles by SEM revealed rounded shaped and/or nearly spherical shapes particles with relatively smooth surfaces and a mean particle diameter between 4 and 12 µm. FT-IR and XRD studies highlighted the ionic nature of the interaction between the protonated amine groups of chitosan and the carboxylate ions of the cross-linking agent and the presence of citric acid embedded in the chitosan microparticles. Importantly, it was demonstrated that the properties of the chitosan microparticles could be modulated by changing the degree of cross-linking of the polymer, which is governed by the chitosan-to-cross-linker mass ratio and the pH of the solution. A range of samples with varying formulation parameters were used to investigate the interaction between chitosan and citric acid. Different physicochemical properties of these microparticles were analyzed to comparatively evaluate the effect of the preparation conditions on their ability to form stable protein delivery systems. The increase in the chitosan-to-citric acid ratio leads to a decrease in D_m_ and erosion of the microparticles. When pH increases, the average size of the samples increases and degradation decreases. Bovine serum albumin was considered as a model drug protein to investigate protein release profiles of the cross-linked microparticles. On the basis of the data obtained in this investigation, different structures of the microparticles with varying degrees of cross-linking of the chitosan were proposed. A non-cross-linked sample of the microparticles showed low efficiency of BSA encapsulation and rapid initial release of the protein caused by a significant destruction of their shells. It was shown that the encapsulation efficiency of biologically active substances in the citric acid cross-linked particles was increased by a factor of 4, when compared with any relevant previous studies [31]. In conclusion, the results presented in this study indicate that the preparation of chitosan microparticles via ionic crosslinking using citric acid is fully dependable on the preparation protocol. The ability to control the percentage of entrapped drug and its release profiles during microparticle preparation is crucial and expected to contribute on developing carriers for the oral protein and peptide drug delivery.

## Supplementary Materials

The following are available online at https://www.mdpi.com/1996-1944/13/8/1989/s1, Figure S1: X-ray diffractograms of the chitosan microparticles: MP5700 and MP5704 (a), MP5706 (b), MP5708, MP5008 and MP4008 (c).
