# Supplementary material for: Modulation of Entrapment Efficiency and In Vitro Release Properties of BSA-Loaded Chitosan Microparticles Cross-Linked with Citric Acid as a Potential Protein–Drug Delivery System
Source: Materials (Basel). 2020 Apr 24;13(8):1989. doi: 10.3390/ma13081989 (PMC7216016; doi:10.3390/ma13081989)
Supplement: Supplementary file 1 [file materials-13-01989-s001.pdf]

materials

MDPI

Article

Modulation of Entrapment Efficiency and *in Vitro* Release Properties of BSA-Loaded Chitosan Microparticles Cross-Linked with Citric Acid as a Potential Protein–Drug Delivery System

Natalia Sedyakina [1], Andrey Kuskov [2,*], Kelly Velonia [3], Nataliya Feldman [1], Sergey Lutsenko [1], and Grigory Avramenko [2]

[1] Department of Biotechnology, I.M. Sechenov First Moscow State Medical University (Sechenov University), Moscow 119991, Russia; nsedyakina@mail.ru (N.S.); n_feldman@mail.ru (N.F.); svlutsenko57@mail.ru (S.L.)

[2] Department of Technology of Chemical Pharmaceutical and Cosmetic Substances, D. Mendeleev University of Chemical Technology of Russia, Moscow 125047, Russia; a_n_kuskov@mail.ru

[3] Department of Materials Science and Technology, University of Crete, Heraklion 70013, Greece; velonia@materials.uoc.gr

* Correspondence: ankuskov@muctr.ru; Tel.: +7-499-972-4808

Received: 8 April 2020; Accepted: 21 April 2020; Published: 24 April 2020

Supplementary Materials:

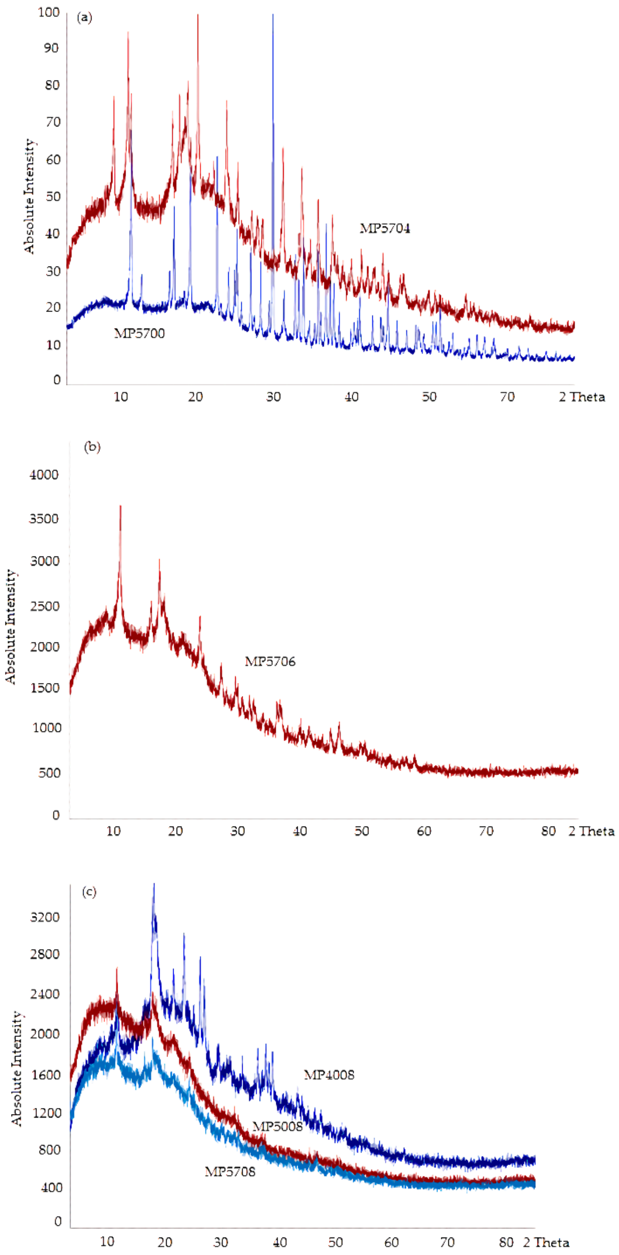

Figure S1. X-ray diffractograms of the chitosan microparticles: MP5700 and MP5704 (**a**), MP5706 (**b**), MP5708, MP5008 and MP4008 (**c**).

© 2020 by the authors. Submitted for possible open access publication under the terms and conditions of the Creative Commons Attribution (CC BY) license (http://creativecommons.org/licenses/by/4.0/).
